# Contributions of cuticle permeability and enzyme detoxification to pyrethroid resistance in the major malaria vector *Anopheles gambiae*

**DOI:** 10.1038/s41598-017-11357-z

**Published:** 2017-09-11

**Authors:** Gildas A. Yahouédo, Fabrice Chandre, Marie Rossignol, Carole Ginibre, Vasileia Balabanidou, Natacha Garcia Albeniz Mendez, Olivier Pigeon, John Vontas, Sylvie Cornelie

**Affiliations:** 1 Institut de Recherche pour le Développement (IRD), Maladies Infectieuses et Vecteurs: Ecologie, Génétique, Evolution et Contrôle (MIVEGEC), UMR - IRD224, CNRS 5290 Montpellier, France; 2 Institute of Molecular Biology and Biotechnology, Foundation for Research and Technology-Hellas, Heraklion, 70013 Greece; 3 Department of Biology, University of Crete, Vassilika Vouton, Heraklion, 70013 Greece; 4 Walloon Agricultural Research Centre (CRA-W), Agriculture and Natural Environment Department (D3), Plant Protection Products and Biocides, Physico-chemistry and Residues Unit (U10), B-5030 Gembloux, Belgium

## Abstract

To tackle the problem of insecticide resistance, all resistance mechanisms need to be studied. This study investigated the involvement of the cuticle in pyrethroid resistance in a strain of *Anopheles gambiae*, MRS, free of kdr mutations. Bioassays revealed MRS to be resistant to pyrethroids and DDT, indicated by increasing knockdown times and resistance ratios. Moreover, biochemical analysis indicated that metabolic resistance based on enhanced CYP450 activity may also play a role. Insecticide penetration assays showed that there were significantly lower amounts of insecticide in the MRS strain than in the susceptible control. Analysis of the levels of the selected transcripts by qPCR showed that CYP6M2, a major pyrethroid metaboliser, CYP4G16, a gene implicated in resistance via its contribution to the biosynthesis of elevated epicuticular hydrocarbons that delay insecticide uptake, and the cuticle genes CPAP3-E and CPLCX1 were upregulated after insecticide exposure. Other metabolic (CYP6P3, GSTe2) and cuticle (CPLCG3, CPRs) genes were also constitutively upregulated. Microscopic analysis showed that the cuticle layers of the MRS strain were significantly thicker than those of the susceptible strain. This study allowed us to assess the contribution made by the cuticle and metabolic mechanisms to pyrethroid resistance in *Anopheles gambiae* without target-site mutations.

## Introduction

The number of insecticides available for use in public health is very limited due to the risks to human health and the environment. Furthermore, public health pesticides are a niche market compared to agriculture, where there is more funding for Research and Development because of concerns over the food chain and food safety. Effective vector control requires mosquito insecticides to be as suitable as possible to the task and to take into account resistance mechanisms that have been less widely investigated but which may have multiple interactions with other mechanisms^1^. Most insecticides are neurotoxic and 80% of them target the synaptic cleft^2^.

The class of pyrethroid insecticides is the most commonly used for public health. For a quarter of a century, insecticide-treated bed nets have been the best and cheapest control tool for individual and community protection against vector-borne diseases, especially malaria^3^. In Africa, the broad coverage of long-lasting insecticidal nets (LLIN) for malaria prevention, in combination with other factors, has reduced *Plasmodium falciparum* transmission by 50%^3^. However, insecticide resistance may compromise the effectiveness of vector control programmes across Africa. The major African malaria vector, *Anopheles gambiae s*.*s*., is widely resistant to pyrethroids^4–6^ and this resistance is grounded in several mechanisms^7, 8^. Two of which have been widely studied: the increase in detoxification processes as a result of high levels of multi-function oxidases (MFO), non-specific esterases (NSE) and glutathione S transferases (GST)^9, 10^, and reduced sensitivity of the target proteins on which the insecticide acts in the voltage-gated sodium channel due to point mutation of the genes encoding these target proteins, i.e., the knockdown resistance (kdr) mutation^11^. Behavioural resistance is still poorly understood, but changes in behaviour associated with resistance mechanisms have occasionally been observed^2, 12^. Furthermore, several studies have reported a change in the biting behaviour of wild *Anopheles* populations after mass distribution of LLINs for vector control^13–15^.

As pyrethroid insecticides act through contact, in order to reach the nervous system they must first cross the mosquito exoskeleton, i.e., the cuticle. At that stage, some insects develop a mechanism that reduces penetration of the insecticide/toxic compound. This resistance mechanism has been demonstrated mainly in agricultural pests^16–18^, but relatively neglected in mosquitoes. However, a previous study suggests a possible association between cuticle thickness and resistance to pyrethroids in *Anopheles funestus* ^19^, while another more recent study found reduced penetration of deltamethrin in resistant *Anopheles gambiae* ^20^. Furthermore, comparative transcriptome analysis has shown significant differences in the transcripts of some cuticular proteins between deltamethrin-resistant and susceptible *Anopheles gambiae* in Kenya^21^. Alteration of the cuticle structure has also been observed alongside insecticide resistance in *Anopheles arabiensis* ^22^. In Southeast Asia, there is probably an association between cuticle protein expression and deltamethrin resistance in *Culex pipiens pallens* ^23^. The cytochrome P450, CYP4G16, consistently over-expressed in pyrethroid-resistant *Anopheles arabiensis* and *Anopheles coluzzii* ^22, 24^, has recently been reported to be involved in cuticular hydrocarbon production in *Anopheles gambiae* ^20^. The cuticle of insects is a natural mechanical barrier against dehydration^25–27^, as well being where pheromones are stored and released and the fixation support of insect muscles^28, 29^. However, its role in insecticide resistance in *Anopheles gambiae* is still unknown. One of challenges in studying cuticular resistance is to isolate the mechanism from target-site resistance and/or metabolic resistance. In this study, we isolated and selected with pyrethroids an *Anopheles gambiae* strain without target site mutations from the Côte d’Ivoire and investigated the relative contributions of cuticular and metabolism-based resistance in the phenotype.

## Methods

### Mosquito strains

The resistant strain of *Anopheles gambiae s*.*s* used in this study, termed MRS without kdr and Ace1 mutations, came from a field resistant population that originated from the M’be strain in Côte d’Ivoire (West Africa) harbouring the L1014F kdr mutation as well as metabolic resistance. The target site mutations, i.e., kdr (L1014F, L1014S, N1575Y) and Ace1 (G119S), were eliminated in MRS in order to exclude their contribution to the phenotype. M’be was selected as it has a lower frequency (0.33) in the kdr allele^30^. To isolate and select MRS, the resistant strain from M’be was crossbred once with the laboratory susceptible reference strain Kisumu. The males from this first progeny (G1) were backcrossed with Kisumu females for a second generation of progeny (G2). The males from G2 were then coupled with Kisumu females so that at the third generation only the progeny of couples of which the male was free of target-site mutations were maintained. This third generation, free of target-site mutations, was termed MRS. The resistance of MRS was maintained in the laboratory by performing adult selection using deltamethrin-impregnated (0.05%) papers according to the WHO test tube protocol^31^ for more than 20 generations. The strain was regularly genotyped by real-time polymerase chain reaction (qPCR) to confirm the absence of target-site mutations.

### Bioassays

The conventional WHO bioassay^31^ was used to test the susceptibility of MRS to six insecticides at diagnostic concentrations: two pyrethroids, deltamethrin (0.05%) and permethrin (0.75%); one pseudo-pyrethroid, etofenprox (0.50%); one carbamate, bendiocarb (0.10%); one organophosphate, fenitrothion (1%); and one organochlorine, DDT (4%). At 2–5 days old, MRS females were exposed to impregnated papers for 1 hour according to the standard WHO bioassay^31^. Susceptible Kisumu females were used to verify the quality of the impregnated papers. The number of mosquitoes knocked down during exposure was regularly recorded to determine KdT_50_, the time point at which 50% of the mosquitoes are knocked down, and KdT_95_, the time point at which 95% of the mosquitoes are knocked down. After 1 hour of exposure, the mosquitoes were transferred to holding tubes without insecticide and fed with a 10% honey solution. Mortalities were recorded 24 h post-exposure. For each test, a batch of 25 MRS females were also exposed to untreated paper as a negative control group, and Abbott’s formula was used to correct the mortality in the treated group when control mortalities exceeded 10%. According to the resistance cut-offs established by the WHO, mortality ≤90% indicates a population resistant to the insecticide, >97% a susceptible population, and 90–97% suspected resistance. After 24 h, control MRS were individually stored at −80 °C for biochemical analysis. Other batches of unexposed MRS from the same population and the 24 h survivors were preserved in RNA extraction buffer (NucleoSpin, MACHEREY – NAGEL) and kept at −80 °C for transcriptome analysis.

Bioassays were also conducted after pre-exposure of mosquitoes to the synergist piperonyl butoxide (PBO), an inhibitor of cytochrome P450 monooxygenase (CYP450). A significant increase in the 24 h mortality rate after PBO exposure before insecticide would indicate the contribution of CYP450 to insecticide resistance. The decrease of resistance level when compared to a susceptible reference strain tested under the same conditions allows to estimate the relative contribution of CYP450 to the overall resistance. To this end, females from Kisumu and MRS strains were exposed to three different treatments in WHO test tubes: 1 h with PBO (4%), 1 h with deltamethrin (0.05%), and 1 h with PBO (4%) followed by 1 h with deltamethrin (0.05%). The number of mosquitoes knocked down during each treatment was recorded at 5, 10, 15 and 20 minutes, then every 10 minutes until 1 h for KdT determination.

### Biochemical assays

Cytochrome P450 monooxygenase levels, and GST and NSE activity were determined using, respectively, tetramethylbenzidine, chlorodinitrobenzene, and α- and β-napthyl acetate substrates, as described in the WHO manual^32^. Absorbances were read by spectrophotometry using a TECAN Sunrise^TM^ microplate absorbance reader. Data layout was managed with the Magellan software version 7.0 coupled with the spectrophotometer. Forty individual MRS females, unexposed to insecticides and previously kept at −80 °C, and thirty-five Kisumu females were used as reference susceptible strain. To avoid any plate effect, females of MRS and Kisumu were assayed on the same 96-well plates. Mosquitoes having replicates with a variation coefficient higher than 15% were discarded in order to avoid differences arising from possible manual errors. None of mosquitoes was ever fed with blood meal.

### qPCR analysis of selected transcripts

Six genes from three detoxification enzyme families (Cytochrome P450 monooxygenase, NSE, GST) were selected from a literature review of microarray and/or functional validation studies^33–37^. Selection was based on two genes by each detoxification enzyme family (Table 3, Supplementary Information). Similarly, twelve genes were also selected from the same studies because of their involvement in cuticle formation (Table 3, Supplementary Information). The expression of these genes was determined by quantitative real-time polymerase chain reaction (qRT-PCR) in females of Kisumu, unexposed MRS and MRS insecticide survivors. All qPCRs were run on a Roche Light Cycler® 480 using SYBR Green.

### Permeability of the MRS cuticle to insecticide

Permeability of MRS and Kisumu cuticles to deltamethrin was tested by topical application of a 10-times lethal concentration 95 (D10LC_95_ = 1.06 ng/mosquito) of the insecticide. LC_95_ was determined with the susceptible reference strain Kisumu. To overcome deltamethrin degradation by P450 oxidases in resistant bodies after penetration, mosquitoes were exposed to impregnated papers of PBO 4% for 1 h before deltamethrin application. Dicyclohexyl phthalate (DP), a stable chemical compound with a similar structure to deltamethrin, was applied at the same dose as D10LC_95_ as a control. D10LC_95_ and DP topical solutions were prepared in a 0.2 µl volume of acetone. Three biological replicate pools of forty 3–5 day old females, non-blood fed, were selected from both strains for the experiment. The mosquitoes were put to sleep with carbon dioxide (CO_2_) and kept anaesthetised on a cold plate. The 6 legs of each mosquito were joined together on a glass slide under a binocular viewer (Leica Wild Binocular) and 0.2 µl of D10LC_95_ or DP was dropped on the tarsi. All topical applications were carried out under the binocular viewer to avoid mistakes. The treated mosquitoes were picked up by the proboscis and put back on the cold plate for 15 minutes (Kisumu KdT_50_ for deltamethrin). All 6 legs were then completely torn from the body. The rest of the bodies were pooled in 15 ml labelled Falcon tubes and stored at −20 °C for gas chromatography analysis. The mosquito tarsi were chosen for topical application instead of the thorax because the legs can be removed at any time to stop contact with the insecticide and to avoid contamination of samples by deltamethrin residue on the surface of the insects.

### Gas chromatography analysis

All Falcon tubes with mosquito bodies previously stored at −20 °C were frozen in liquid nitrogen for 20 seconds and finely ground glass was added to the contents. 100 µl of a mixture of alpha cypermethrin and lambda-cyhalothrin solutions (internal standards, Sigma-Aldrich) and 900 µl of a 90/10 isooctane/toluene mixture were added (Sigma Aldrich). The mosquito bodies were submitted to extraction by oscillo-vibration (25 movements per second) for 10 minutes. They were then frozen again in liquid nitrogen for 10 seconds, after which further extraction was performed by oscillo-vibration (25 movements per second) for 10 minutes. The extract was filtered with a 0.2 μm nylon filter before the deltamethrin, PBO and DP analyses (Supplementary Information).

### Fixation for microscopic analysis of MRS cuticles

All the mosquitoes subjected to microscopic analysis had the same body size as measured by wing size^38^ as a proxy for selection. Two batches of 10 MRS females and two batches of 10 Kisumu females (3–5 days old, not blood fed) were selected for the experiment. They were very carefully transferred to the bottom of a 1.5 ml Eppendorf tube, which was then completely filled with a fixative solution consisting of 2% glutaraldehyde, 2% PFA in a 0.1 M sodium cacodylate buffer (SCB).

### Transmission Electron microscopy

The electron microscopy protocol was the same as that described by Balabanidou^20^. From observation of the pictures we measured the whole cuticle thickness from the inner layer (endocuticle) to the external layer (exocuticle) with Image J 1.49. The epicuticle layer was sanded down during multiple hexane washes and consequently did not appear on the pictures. The thicknesses of the different layers (endocuticle, mesocuticle and exocuticle) were also measured separately where picture sharpness was sufficient to distinguish between them. Three to five different partition pictures were taken per leg cross-section.

### Statistical analysis

Johnson script^39^ basically set for LC_50_ and LC_95_ determination was modified to R script BioAssay 6.2 (http://www.isem.univmontp2.fr/recherche/equipes/evolution-vecteurs-adaptation-et-symbioses/personnel/labbe-pierrick/) for KdT_50_ and KdT_95_ determination with a 95% confidence interval. The script created a generalized linear model (GLM) with binomial errors in the response variable. A probit link was then fitted to the data where the probit knockdown was a function of the logarithm of times for each strain. This allowed RR_50_ and RR_95_ to be computed and comparisons between two or more populations to be carried out. The levels of enzymatic activity in Kisumu and MRS were compared using a Mann-Whitney test. Relative expressions of target genes were determined according to the ΔΔCt methods described by Schmittgen & Livak^40^ and implemented in the Light Cycler® 480. A Mann-Whitney test was also used to compare the fold changes between the MRS unexposed to insecticides and MRS survivors. The difference between the cuticle thicknesses of Kisumu and MRS, and the penetration of deltamethrin and DP were analysed using a Wilcoxon rank sum test. All statistical analyses were performed with the R statistical software^41^ and differences were considered significant with a *p*-value < 0.05. The graphs were drawn with the ggplot2 package^42^ implemented in R and GraphPad Prism^43^ for Windows.

## Results

### Isolation and characterisation of non-target-site pyrethroid resistance

The resistant strain named MRS was derived from 52 couples (out of 102) of which both parents were free of kdr and Ace1 alleles. MRS mosquitoes were exposed to diagnostic concentrations of six insecticides (2 pyrethroids, 1 pseudo-pyrethroid,1 organochlorine, 1 organophosphate, 1 carbamate) using WHO test kits (Table 1). Mortalities in Kisumu females were always 100%. According to WHO criteria based on 24 h mortality^31^, MRS was resistant to deltamethrin (60.8%), etofenprox (47.0%) and DDT (73.8%), suspected resistant to permethrin (91.9%), and susceptible to fenitrothion (100%) and bendiocarb (100%). Knockdown times in the MRS strain were significantly greater with DDT, the pyrethroids and the pseudo-pyrethroid (Table 1). The use of PBO as a P450 inhibitor did not reduce the knockdown times indicating that these detoxification enzymes probably have a limited role in this effect. However, the 24 h mortality of MRS pre-exposed to PBO was more than 94% suggesting that CYP could act later, after penetration, to metabolise deltamethrin.

**Table 1:** Effects of different insecticides on MRS adults.

Insecticide	Strain	N	KdT_50_ (CI_95_)	KdT_95_ (CI_95_)	Slope (SE)	RR_50_ (CI_95_)	RR_95_ (CI_95_)	24 h Mortality (CI_95_)
Permethrin 0.75%	MRS	96	29.4 (27.5–31.3)	73.3 (65.3–84.8)	4.1 (0.3)	**2.1 (1.1–4.1)**	**2.9 (1.1–7.5)**	91.9 (85.6–97.0)%
	Kisumu	22	14.2 (12.4–18)	25.7 (21.8–34.2)	6.4 (1.1)	—	—	100%
Etofenprox 0.50%	MRS	93	66.7 (60.3–77.7)	151.3 (117.9–228.3)	4.6 (0.6)	**3.4 (1.8–6.5)**	**4.7 (1.6–13.6)**	47.0 (36.9–57.9)%
	Kisumu	25	19.4 (17.5–21.4)	32.3 (28.1–40.4)	7.4 (1.1)	—	—	100%
DDT 4%	MRS	96	53.2 (49.7–57.8)	114.7 (97.1–146)	4.9 (0.5)	**3 (1.5–5.9)**	**4.4 (1.3–14.4)**	73.8 (64.0–82.4)%
	Kisumu	25	17.6 (16.1–19.2)	26.1 (13.1–33.1)	9.6 (1.7)	—	—	100%
Bendiocarb 0.1%	MRS	95	24.1 (22.6–25.7)	42.5 (38.7–47.8)	6.7 (0.5)	2.4 (0.8–7.3)	2.9 (0.7–13.1)	100%
	Kisumu	25	9.9 (8.6–10.9)	14.4 (12.7–19.2)	10.2 (2.5)	—	—	100%
Fenitrothion 1%	MRS	98	no Kd	no Kd	NA	NA	NA	100%
	Kisumu	23	no Kd	no Kd	NA	NA	NA	100%
Deltamethrin 0.05%	MRS	94	35.4 (31.9–39.3)	74.3 (62.5–97.0)	5.1 (0.5)	**2.3 (1.4–3.8)**	**2.7 (1.3–5.7)**	60.8 (50.0–70.6)%
	Kisumu	24	15.3 (14.1–16.5)	27.4 (24.4–32.2)	6.5 (0.7)	—	—	100%
PBO 4% + Deltamethrin 0.05%	MRS	53	23.2 (20.9–25.7)	34.8 (30.5–43.7)	9.4 (1.2)	**2.5 (1.2–5.2)**	2.1 (0.8–5.2)	94.3 (84.3–98.8)%
	Kisumu	52	9.3 (6.3–11.8)	16.9 (12.9–35.2)	6.3 (1.5)	—	—	100%

The 50% and 95% knockdown times (KdT) of MRS and Kisumu with associated 95% confidence intervals (CI_95_). The resistance ratios (RR) 50% and 95% are those of MRS KdT to Kisumu KdT. KdTs were determined by glm with binomial error and probit link function. SE is the standard error associated with the slope of the model, N the number of female adults tested. Significant resistance to the knockdown effect in MRS compared with Kisumu is shown in bold and indicated by an RR > 1 with its corresponding CI_95_, which excluded 1. 24 h mortality <90% indicates resistance to the corresponding insecticide, 90 to 97% suspected resistance, >97% susceptibility^31^.

### Detoxification enzyme activity in MRS resistance

**Figure 1:**
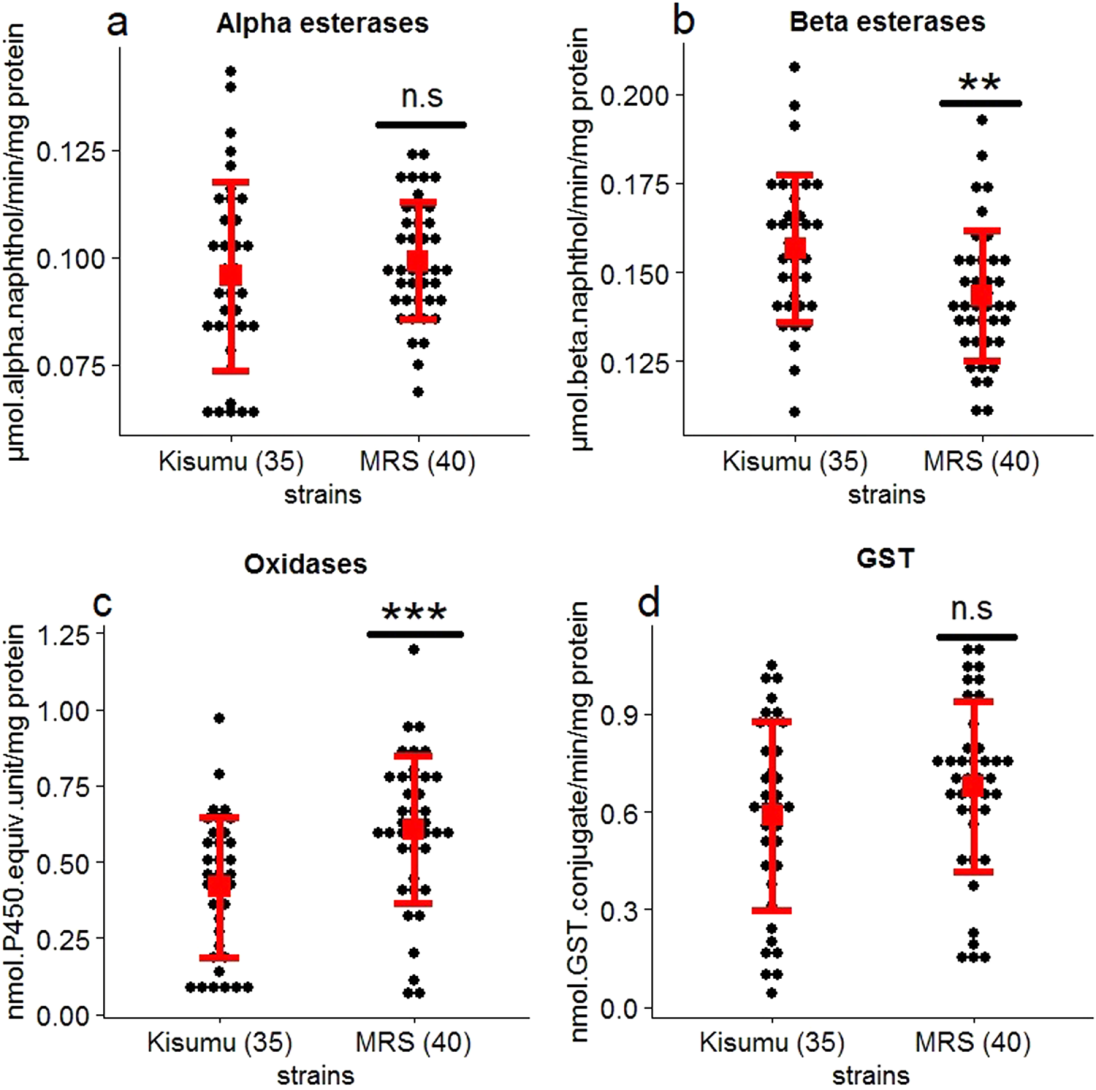
Activity profiles of non-specific alpha (**a**) and beta (**b**) esterases, mixed-function oxidases of cytochrome P450 (**c**) and glutathione S-transferase (GST) (**d**) in susceptible (Kisumu) and resistant (MRS) *An*. *gambiae*. Dots indicate the measured data for all samples, error bars represent means ± standard deviation. Numbers in brackets indicate the number of mosquitoes tested. Enzyme activities were determined by spectrophotometric measurements based on optical densities, and the two strains were compared using the Mann-Whitney U-test. *p-value < 0.05, **p-value < 0.01, ***p-value < 0.001, n.s. = non-significant difference.

Biochemical analysis was conducted to evidence enzyme families involved in MRS resistance to insecticides. The results showed a significant increase in the amount of cytochrome P450 monooxygenase in MRS (0.61 nmol P450 equivalent unit/mg protein) compared with Kisumu (0.42) (W = 387, *p* < 0.001, Fig. 1). Beta esterase activity was significantly lower in MRS (0.14 μmol beta naphthol/min/mg protein) than in Kisumu (0.16) (W = 970, *p* = 0.003, Fig. 1). No significant differences were detected in alpha esterases (W = 597, *p* = 0.28) and GST (W = 571.5, *p* = 0.17). These biochemical results indicate a possible role of cytochrome P450 in the resistance phenotype of the MRS strain, in line with the bioassay results.

### Levels of selected cytochrome P450 and cuticle gene transcripts in MRS

The relative expressions of these genes were determined in MRS unexposed to insecticides (C), MRS 24 h insecticide survivors (R) and the susceptible Kisumu strain (S). A side-by-side comparison (R-C, R-S, C-S) of these relative gene expressions was performed for each insecticide. Only significant (*p* < 0.05) fold changes (Fc > 1) are discussed in this section.

**Figure 2:**
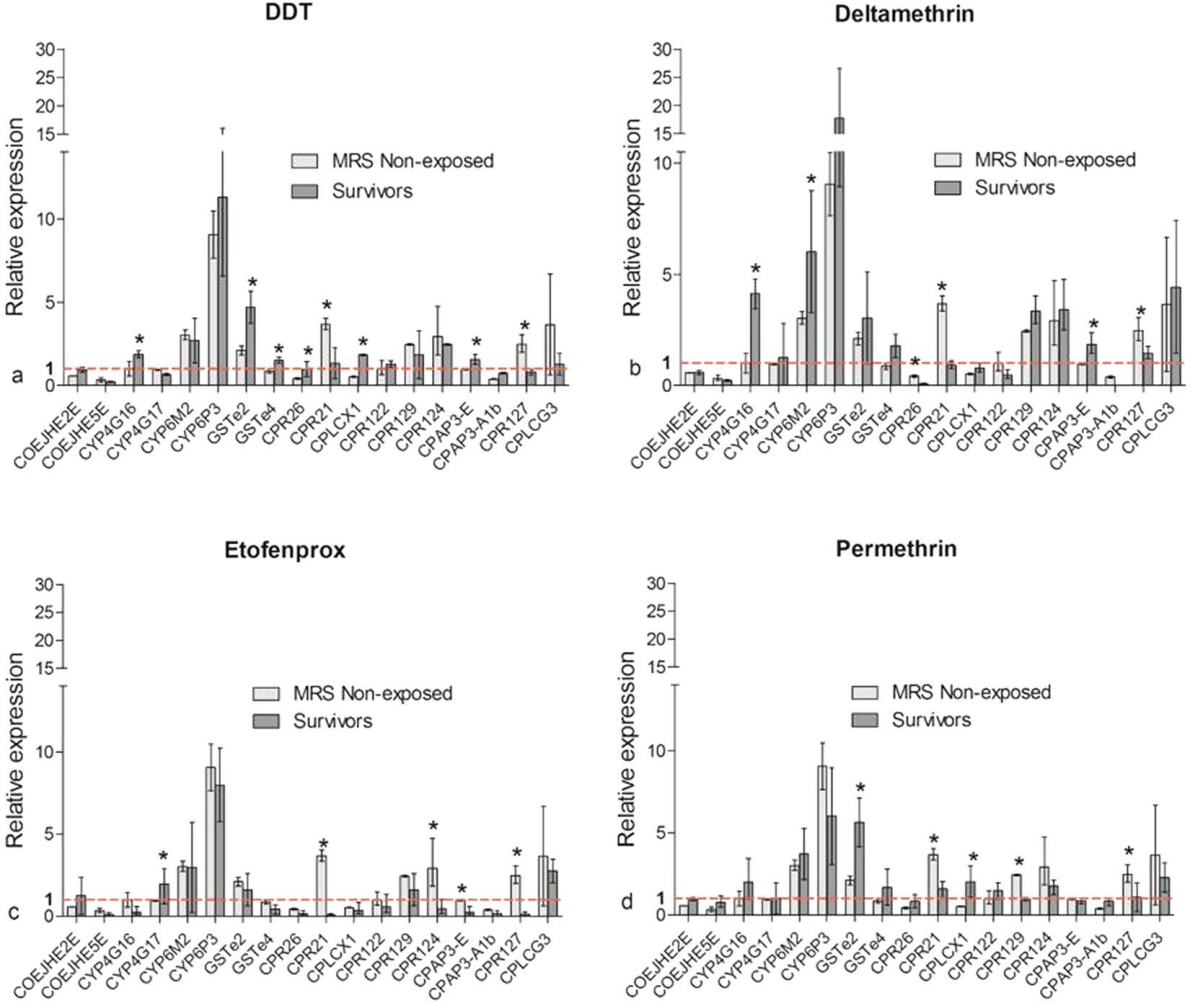
Relative expressions of detoxification and cuticle genes in MRS unexposed to and survivors of DDT (**a**), Deltamethrin (**b**), Etofenprox (**c**) and Permethrin (**d**). Bar plots represent the mean fold changes and error bars the standard deviations. Fold changes were determined by the ΔΔCt method with the susceptible reference Kisumu as calibrator. The bars above the dotted line (fold change = 1) represent upregulation, those under downregulation. The fold changes from three biological replicates and two independent experiments of both groups were compared using the Mann-Whitney U-test. *Significant difference (*p*-value < 0.05).

Specifically, three CYP (CYP6M2, CYP4G16, CYP4G17), two GST (GSTe2, GSTe4) and two cuticle (CPAP3-E, CPLCX1) genes were overexpressed in MRS survivors of the pyrethroids and DDT. The Fc of CYP6M2 ranged from 2.7 to 6.0, CYP4G 1.9 to 4.1 and GST 1.5 to 5.6, while the Fc of both cuticle genes ranged from 1.7 to 2.4 (Fig. 2). In addition, one metabolic (CYP6P3) and four cuticle (CPLCG3, CPR124, CPR129, CPR127) genes were constitutively overexpressed in MRS resistance to insecticides, CYP6P3 having an Fc of 17.8 and CPLCG3 an Fc of 4.4.

### Reduced deltamethrin penetration of the MRS cuticle

The results showed that the MRS cuticle significantly reduced (W = 36, *p* = 0.002) deltamethrin penetration inside the body. Mean deltamethrin content was 0.007 ng/MRS mosquito body, but 0.048 ng/Kisumu mosquito body (Table 2). Where mosquitoes were exposed to PBO before deltamethrin application, mean deltamethrin content was 0.010 ng/MRS body, significant lower (W = 36, *p* = 0.002) than in Kisumu, which was 0.066 ng/body. DP penetration was also significantly reduced (W = 36, *p* = 0.004) by the MRS cuticle. Mean DP content was 0.006 ng/MRS body, but 0.073 ng/Kisumu body (Table 2). In the case of pre-exposure to PBO, the amounts of deltamethrin were substantially higher than in the other experimental conditions, suggesting that PBO either accelerated deltamethrin penetration in the insects or promptly inhibited the action of enzymes metabolising deltamethrin.

**Table 2:** Reduced penetration of deltamethrin in resistant MRS versus susceptible Kisumu after topical application.

Treatment	Sample bodies	ng/mosquito (SD)	*p*-value (Wilcoxon rank)
Deltamethrin	Kisumu	0.048 (0.026)	0.002 (W = 36)
	MRS	0.007 (0.001)	
PBO + Deltamethrin	Kisumu	0.066 (0.136)	0.006 (W = 36)
	MRS	0.010 (0.023)	
Dicyclohexyl phthalate	Kisumu	0.073 (0.039)	0.004 (W = 36)
	MRS	0.006 (0.002)	

Three biological replicate pools of 40 mosquitoes per treatment were used for chemical analysis. Deltamethrin was determined using Gas Chromatography coupled to an Electron Capture Detector (GC-ECD), PBO and dicyclohexyl phthalate by Gas Chromatography with Mass Spectrometry (GC-MS). Dicyclohexyl phthalate was used as control since it has a similar structure to deltamethrin and is not metabolised by the insects. The figures represent the means with standard deviations (SD) of the chemical contents. ng = nanogram. *p*-value < 0.05 indicates a significant difference according to the Wilcoxon rank sum test.

### Enriched cuticle thickness in MRS

**Figure 3:**
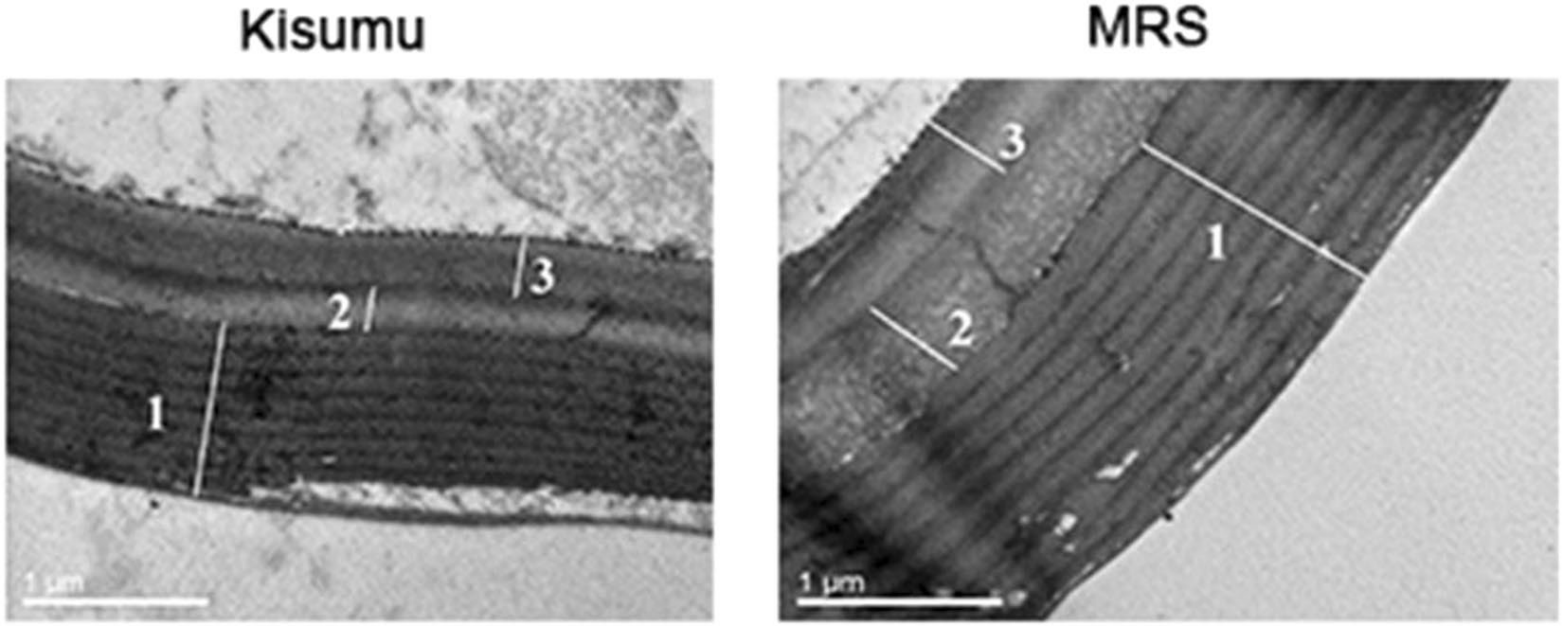
Cuticle ultrastructure in susceptible Kisumu and resistant MRS. Cross sections of the apical region of the mosquitoes’ femur. (1) Exocuticle, (2) Mesocuticle, (3) Endocuticle. The images were taken with a high-resolution transmission electron microscope, model JEM 2–100, at an operating voltage of 80 kV.

**Figure 4:**
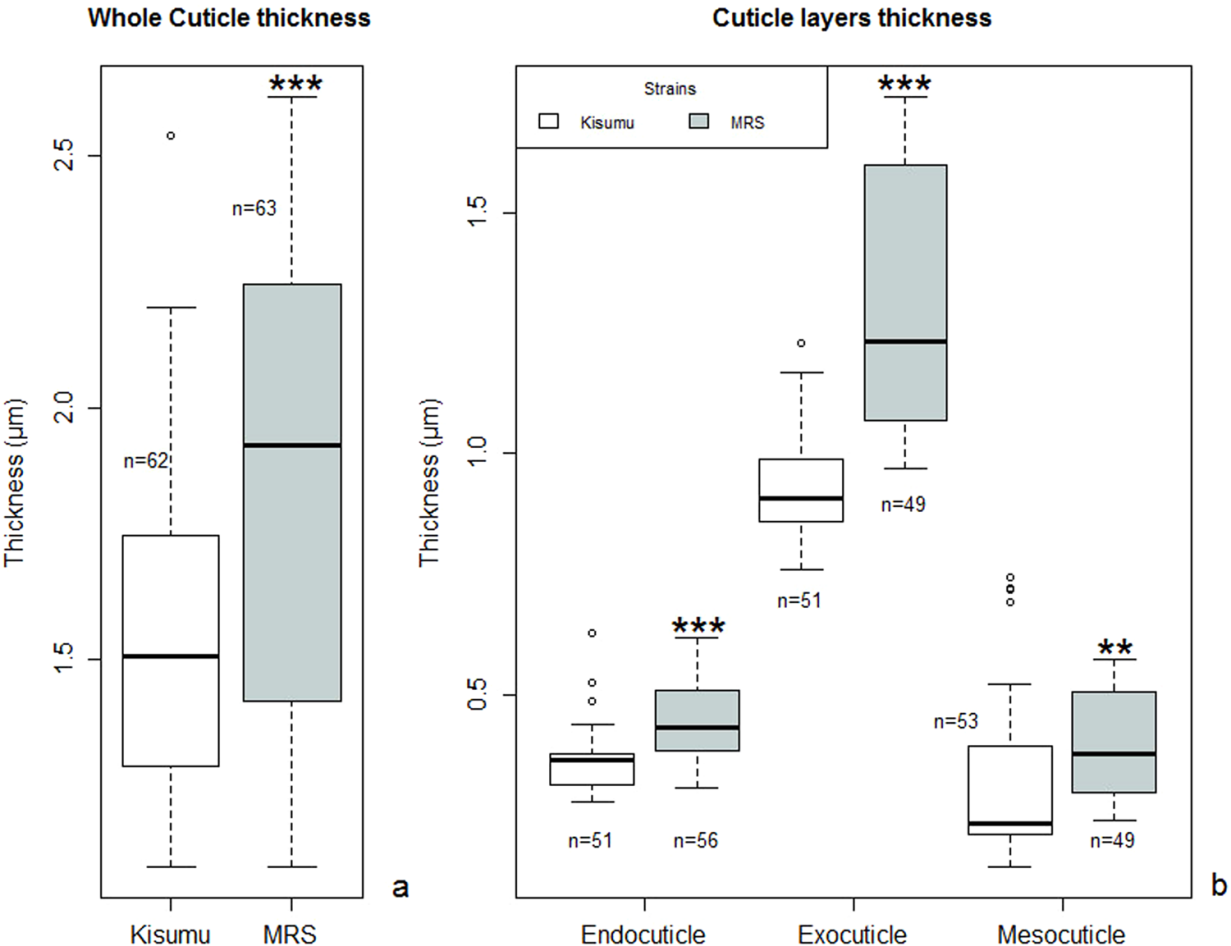
Cuticle thickness in resistant MRS and susceptible Kisumu measured by transmission electron microscope. Thicknesses of the whole cuticle (**a**) and of its various layers (**b**) were measured with an Image J 1.49. Box plots represent the minimum and maximum quartiles, dots the outlying data. n = the number of measures carried out on each batch of 20 mosquitoes (Kisumu & MRS). *p-value < 0.05, **p-value < 0.01, ***p-value < 0.001 according to a Wilcoxon rank sum test.

The mean thickness of the whole MRS cuticle (1.85 ± 0.21 µm) was significantly greater (W = 1197, *p* = 0.0001) than that of Kisumu (1.53 ± 0.11 µm) (Figs 3 and 4). It is worth noting that all the MRS procuticle layers (exo, meso and endo) were significantly thicker than the corresponding Kisumu layers (Figs 3 and 4).

## Discussion

MRS showed resistance to pyrethroid and pseudo-pyrethroid despite the absence of the knockdown resistance (kdr) mutation, a point mutation that confers cross-resistance to pyrethroid and DDT^44^. As MRS was free of kdr, the knockdown time ratios were not expected to significantly increase where only metabolic resistance is involved^45, 46^. In our study, the knockdown time ratio of MRS was not change after the pre-exposure to the CYP450 inhibitor PBO and was higher than 2. It means that MRS was more than twice resistant to the knockdown effect of deltamethrin compared to the susceptible reference strain Kisumu, even after the use of PBO. The similarity of knockdown times with or without PBO confirmed that CYP450 did not protect MRS against the knockdown but rather against lethal effect of deltamethrin, a view supported by the significant increase in the 24 h mortality rate after the use of PBO. The increase in knockdown times, in absence of the kdr mutation with the non-involvement of CYP450, might be due to slower penetration of the MRS cuticle by the insecticide, thereby delaying transport to the nervous system.

Interesting results emerged from experiments on deltamethrin penetration, which support the hypothesis that cuticular mechanisms are involved in MRS resistance and have a direct impact on insecticide penetration. Pyrethroid insecticides for mosquito control in public health act by contact and, regardless of the molecule structure, they need to penetrate the insect to reach their target, the cuticle being the first barrier to penetration. The results show that the MRS cuticle significantly reduced deltamethrin penetration, and test with dicyclohexyl phthalate confirmed this. Topical application of deltamethrin to mosquito tarsi instead of the thorax has two advantages: firstly, it better mimics real contact in natural conditions because mosquitoes use their tarsi to land on impregnated supports (LLINs, sprayed walls, etc.); secondly, whole mosquito legs can be removed from the body to prevent deltamethrin penetration at a given time and to avoid contamination of samples by deltamethrin remaining on the surface of the insects. In addition, gas chromatography identified only those deltamethrin molecules that crossed the cuticle and were present inside the mosquito body. This is the first time that such topical applications have been used with cold techniques. A direct consequence of lower insecticide penetration could be greater efficacy of metabolic processes.

The biochemical results also indicated an association between higher cytochrome P450s and MRS resistance to pyrethroids, and showed that neither non-specific esterase nor GST activities were significantly higher in MRS. Analysis of selected gene transcripts by qPCR showed that CYP6M2 and CYP6P3 were constitutively upregulated in MRS. Both genes are regularly found overexpressed and associated with pyrethroid resistance in wild *Anopheles gambiae* populations from West Africa^24, 47–49^. CYP6M2 can metabolise pyrethroids and insecticides from other families, such as DDT, *in vitro* ^50, 51^ and CYP6P3 is also known to metabolise pyrethroids as well as bendiocarb *in vitro* ^37, 52^. Transcriptome analysis confirmed the biochemical results except for GST, since GSTe2 was upregulated in MRS even though regulation was lower than for CYP6. Biochemical analysis is not always reliable for measuring GST activity in *Anopheles gambiae*, since specific activities with the substrate chlorodinitrobenzene are lower for epsilon GSTs like GSTe2 than those reported for other insect delta class GSTs^53^. An interesting result was the overexpression of CPAP3-E and CYP4G16 in survivors of deltamethrin exposure compared to unexposed mosquitoes. The potential ortholog of CYP4G16 in *Drosophila melanogaster*, CYP4G1, catalyses the final step of cuticular hydrocarbon biosynthesis^54^, and CYP4G16 has also been found to be overexpressed in resistant *Anopheles coluzzii* ^24^. A recent study on resistant *Anopheles gambiae* showed that CYP4G16 was highly abundant in the oenocytes, the insect cell type thought to secrete hydrocarbons^20^. Overexpression of CYP4G16 in our study might be associated with cuticle reinforcement in resistance. CPAP3-E is one of seven members of the CPAP3 family of proteins (cuticular proteins analogous to peritrophins with three chitin binding domains) in *Anopheles gambiae* ^55^. CPAP3 function in *Anopheles gambiae* is unknown but in *Drosophila melanogaster* there is one orthologue of CPAP3-E (obstructor-E). In *Drosophila melanogaster*, obstructor-E regulates the arrangement of chitin and directs the formation of supracellular ridges on the larval cuticle. It affects the body shape by controlling the mechanic property of the exoskeleton^56^. In this study, CPAP3-E was also found to be overexpressed in survivors of DDT but not in survivors of permethrin and etofenprox exposure. These differences in the expression of CPAP3-E according to insecticide suggest a role in resistance mechanism in MRS and may be specific to some insecticide molecules. It is important to specify that CPAP3s are present in all insect orders^57^. Altogether, they play essential roles in the structure of insect cuticle. Moreover, CPLCX1 (unclassified cuticular protein) was also found to be overexpressed in survivors of DDT and permethrin, suggesting that it is implicated in MRS resistance. It was identified from proteomic analysis of *Anopheles gambiae* cast cuticles, but its function is unknown^58^. The fact that it was overexpressed in survivors of deltamethrin exposure suggests it is potentially involved in resistance. Apart from CPAP3-E and CPLCX1, the other four cuticle genes, CPLCG3, CPR124, CPR129 and CPR127, were constitutively overexpressed in MRS and probably contributed to cuticular resistance. In a previous study, CPLCG3 was twice overexpressed in pyrethroid-resistant compared to pyrethroid- susceptible *Anopheles gambiae* ^59, 60^. The product of this gene is a structural protein of the endocuticle layer participating in cuticle thickness^61^. Further investigation, using RNA interference, for example, should reveal their relative contributions to resistance phenotypes.

The cuticle is a stack of layers consisting of different proteins and chitin. As cuticle genes were overexpressed in MRS, the expectation would be a thickening of the whole cuticle, which was confirmed by microscopic analysis that showed it to be significantly thicker than that of the susceptible strain. Furthermore, every procuticle layer in MRS was thicker than in Kisumu and the outer procuticle layer was the thickest in both strains. Fluorescence *in situ* hybridization would allow overexpressed proteins from cuticle genes to be localised to make sure they belonged to at least one of three cuticle layers. None of the cuticular proteins necessarily belongs to the cuticle since some of them have been found in Johnston’s organ and the corneal lens of *Anopheles gambiae* using EM immunolocalization^62^. In addition, some cuticle proteins with chitin-binding domain 2 belong to the procuticle and also to the midgut chitinous peritrophic matrix in *Drosophila melanogaster* ^63^.

The molecular and physiological experiments carried out in this study provide strong evidence of cuticle involvement in resistance mechanisms in *Anopheles gambiae* MRS. MRS resistance to deltamethrin was both metabolic-based and cuticular, but it was difficult to separate the two mechanisms, which would enable their relative contributions to resistance to be determined. The cuticular mechanism mainly acts in concert with CYPs, but not all the actors involved are known. Further whole transcriptome analyses, such as RNA sequencing and RNA interference, would be useful to identify and select other cuticle gene candidates involved in cuticular resistance.

## Electronic supplementary material

Contributions of cuticle permeability and enzyme detoxification to pyrethroid resistance in the major malaria vector Anopheles gambiae.
