# Author Correction: Contributions of cuticle permeability and enzyme detoxification to pyrethroid resistance in the major malaria vector *Anopheles gambiae*

**DOI:** 10.1038/s41598-018-24094-8

**Published:** 2018-04-12

**Authors:** Gildas A. Yahouédo, Fabrice Chandre, Marie Rossignol, Carole Ginibre, Vasileia Balabanidou, Natacha Garcia Albeniz Mendez, Olivier Pigeon, John Vontas, Sylvie Cornelie

**Affiliations:** 1 Institut de Recherche pour le Développement (IRD), Maladies Infectieuses et Vecteurs: Ecologie, Génétique, Evolution et Contrôle (MIVEGEC), UMR - IRD224, CNRS 5290 Montpellier, France; 2 Institute of Molecular Biology and Biotechnology, Foundation for Research and Technology-Hellas, Heraklion, 70013 Greece; 3 Department of Biology, University of Crete, Vassilika Vouton, Heraklion, 70013 Greece; 4 Walloon Agricultural Research Centre (CRA-W), Agriculture and Natural Environment Department (D3), Plant Protection Products and Biocides, Physico-chemistry and Residues Unit (U10), B-5030 Gembloux, Belgium

Correction to: *Scientific Reports* 10.1038/s41598-017-11357-z, published online 11 September 2017

The Acknowledgements section in this article is incorrect.

“G.A.Y. was supported by the Mediterranean Infection Foundation. We thank the LabEx CeMEB for supporting G.A.Y.’s Scientific Immersion at Heraklion, and INFRAVEC2 for funding the cuticular analysis. Very special thanks go to Philippe Clair for excellent advice on qPCR, the Treilles foundation:« La fondation des treilles, créée par Anne Gruner Schlumberger, a notamment pour vocation d’ouvrir et de nourrir le dialogue entre les sciences et les arts afin de faire progresser la création et la recherche contemporaines. Elle accueille également des chercheurs et des écrivains dans le domaine des Treilles (Var) www.les-treilles.com ». V.B. was supported by a Scholarship for Strengthening Post-Doctoral Research from The Greek State Scholarships Foundation (IKY) within the framework of the Operational Programme “Human Resources Development Program, Education and Life-Long Learning”. This project is co-financed by the European Social Fund-ESF and the Greek government.”

should read:

“Very special thanks go to Philippe Clair for excellent advice on qPCR. Mosquitoes were reared and tested thanks to the technical platform dedicated to vectors at IRD. This platform is a member of the Vectopole Sud network and of the sharing facilities from the LabEX CEMEB (Centre Méditerranéen de l’Environnement et de la Biodiversité) in Montpellier. G.A.Y. was supported by the Mediterranean Infection Foundation, the LabEx CeMEB for scientific immersion at Heraklion, and INFRAVEC2 for funding the cuticular analysis. V.B. was supported by a Scholarship for Strengthening Post-Doctoral Research from The Greek State Scholarships Foundation (IKY) within the framework of the Operational Programme “Human Resources Development Program, Education and Life-Long Learning”. This project is co-financed by the European Social Fund-ESF and the Greek government.”

