# Supplementary material for: Contributions of cuticle permeability and enzyme detoxification to pyrethroid resistance in the major malaria vector Anopheles gambiae
Source: Sci Rep. 2017 Sep 11;7:11091. doi: 10.1038/s41598-017-11357-z (PMC5593880; doi:10.1038/s41598-017-11357-z)
Supplement: Supplementary file 1 — Contributions of cuticle permeability and enzyme detoxification to pyrethroid resistance in the major malaria vector Anopheles gambiae. [file 41598_2017_11357_MOESM1_ESM.pdf]

Contributions of cuticle permeability and enzyme detoxification to pyrethroid resistance in the major malaria vector *Anopheles gambiae.*

Gildas A. Yahouédo[1*], Fabrice Chandre[1], Marie Rossignol[1], Carole Ginibre[1], Vasileia Balabanidou[2], Natacha Garcia Albeniz Mendez[3], Olivier Pigeon[3], John Vontas[2], Sylvie Cornelie[1].

Affiliations

[1]Institut de Recherche pour le Developpement (IRD), Maladies Infectieuses et Vecteurs: Ecologie, Genetique, Evolution et Contrôle (MIVEGEC), UMR - IRD224 - CNRS 5290, Montpellier, France.

[2]Institute of Molecular Biology and Biotechnology, Foundation for Research and Technology-Hellas, Heraklion, 70013, Greece. Department of Biology, University of Crete, Vassilika Vouton, Heraklion, 70013, Greece.

[3]Walloon Agricultural Research Centre (CRA-W), Agriculture and Natural Environment Department (D3), Plant Protection Products and Biocides Physico-chemistry and Residues Unit (U10), B-5030 Gembloux, Belgium.

[*] Corresponding author: Gildas A. YAHOUEDO (gildasy@gmail.com)

Supplementary methods

Transcriptomic analysis of MRS

The total mRNA of Kisumu, MRS non-exposed and MRS survivors were extracted by pools of five mosquitoes using NucleoSpin RNA kit (MACHEREY – NAGEL) according to the manufacturer's instructions. The integrity of RNA was checked using Agilent 2100 Bioanalyzer (Agilent technologies). The Super Script III Reverse Transcriptase ™ kit (Life Technologies) was used to synthesize the first strand cDNA from 400 ng of total RNA according to manufacturer recommendations. The Kisumu females were used as calibrator and the ribosomal genes Rsp7 and Rpl8 as housekeeping gene (shown to be consistent and with no differential expression between susceptible and resistant)[1]. Three biological and technical replicates were run for each sample. The primers used were designed on NCBI

(http://www.ncbi.nlm.nih.gov/tools/primer-blast/) and qPCR was run on Roche Light Cycler® 480 using the SYBR Green Master Mix. The standard curves were generated using five times serially diluted cDNA sample to assess the PCR efficiency. The PCR efficiency criterion was 100 ± 10 % for all of the genes and a single melting curve peak indicating the specificity. The cycling programs were 95 °C for 10min hot start and 40 cycles of 95 °C for 10s, 68 °C for 10s and 72 °C for 10s.

Gas chromatography analysis

Deltamethrin was determined by gas chromatography coupled to an electron capture detector (GC-ECD), PBO and DP by gas chromatography coupled to a mass spectrometer (GC-MS) detector, and all three were quantified by internal calibration. The contents of the active substance and synergist were calculated using a calibration line based on reference solutions of deltamethrin, PBO and DP at different concentrations and containing known concentrations in internal standards to correct the matrix effect. Before analysing the samples, the analytical methods by GC-ECD and GC-MS were successfully validated for their specificity, matrix effect, linearity of chromatographic response, accuracy, repeatability and limit of quantification (LOQ).

Reference

1. Djouaka, R. F. *et al.* Expression of the cytochrome P450s, CYP6P3 and CYP6M2 are significantly elevated in multiple pyrethroid resistant populations of Anopheles gambiae s.s. from Southern Benin and Nigeria. *BMC Genomics* **9,** (2008).

Table 3: Target and reference genes with the corresponding primers and efficiencies. The genes involved in the cuticle formation have a grey background, those belonging to the detoxification enzyme families have a white background in the target genes category.

	N° Accession vectorbase	Gene name	Sens	Primer sequences (5’−3’)	Efficiency
**Target genes**	AGAP006001	CPR26	FW	CGCTGGATGACTCCCGAAAT	1.9
			RV	ATCGCCTCATTATCCGTGCC	
	AGAP005996	CPR21	FW	ATTGCTTTCGCTTTCGTCGT	1.97
			RV	TCAGGATCTGGGCGTTCTTG	
	AGAP001329	CPLCX1	FW	GTGATTGCCTTCGCTCTGTG	1.9
			RV	TACTTAGCCACTGCTGGTGC	
	AGAP003384	CPR122	FW	TTGAATACACTGCCGACCCG	1.9
			RV	GCCTTTACCACACCAGCTCC	
	AGAP000085	CPR129	FW	ACCAAGGCAAGGGCAAGTTT	1.9
			RV	ATGGTAGCCAAACTCGTCGG	
	AGAP003390	CPR124	FW	CCGAGTTCACCGATGCCTAC	2.01
			RV	CTCCTGCGTCTTGGAGTCAC	
	AGAP009405	CPAP3-E	FW	CGGCTACTACAAGGTCGGTG	1.95
			RV	GGGGCAGTCAAGGACATAGG	
	AGAP000987	CPAP3-A1b	FW	ACTTCGAGTTTACCTGCCCG	1.9
			RV	TTGAACACCTGGTCGCACTT	
	AGAP000344	CPR127	FW	GGCGGACGGTACGTTTAAGA	1.9
			RV	GTACCCTGAGGCTCGAAACC	
	AGAP008446	CLPCG 3	FW	CACGCCGCCATCCACGCTGCTT	1.89
			RV	GGCAGGGGCGCGACATGCACAG	
	AGAP001076	CYP4G16	FW	ATGGCGTCAACATCGCAAAC	1.9
			RV	TCGCACATTTTCATCACGGC	
	AGAP000877	CYP4G17	FW	GCTACATCCCGTTCAGTGCT	1.9
			RV	TCCTTCTCGGTCAGGTTGGA	
	AGAP008212	CYP6M2	FW	CTGGCGTTGAATCCAGAGGT	2
			RV	CGGGGACGTGGTAGTCTTTC	
	AGAP002865	CYP6P3	FW	GACGTGCGCGTTCGGCATCGAGT	1.89
			RV	CGTCGGGTAGGACGACGCGAGGA	
	AGAP009193	GSTe4	FW	AGCCACGCCATCAATGTGTA	1.95
			RV	ACGCCCGAATCGAAGTGTAG	
	AGAP009194	GSTe2	FW	GCCGGAATTTGTGAAGCTAAACCCG	1.97
			RV	TGCTTGACGGGGTCTTTCGGAT	
	AGAP005837	COEJHE5E	FW	TGAATGTGTACCGCCCCAAA	1.93
			RV	AAGCCTAACACACCGAGACG	
	AGAP005834	COEJHE2E	FW	GGGCGTTAGGCAGTACATCA	1.92
			RV	GGCTGGCGGAACAGATAGTT	
**Reference genes**	AGAP010592	Rps7	FW	ATTGCCGAGCGCCGCATTCT	1.96
			RV	GACGCGGATACGCTTGCCGA	
	AGAP005802	Rpl8	FW	CCGTGCGTACCACAAGTACA	1.93
			RV	CAGCGATGAGACCGACCTTG	
